# Methyl jasmonate enhances ursolic, oleanolic and rosmarinic acid production and sucrose induced biomass accumulation, in hairy roots of *Lepechinia caulescens*

**DOI:** 10.7717/peerj.11279

**Published:** 2021-04-27

**Authors:** Victor M. Vergara-Martínez, Samuel E. Estrada-Soto, Susana Valencia-Díaz, Karlina Garcia-Sosa, Luis Manuel Peña-Rodríguez, José de Jesús Arellano-García, Irene Perea-Arango

**Affiliations:** 1 Centro de Investigación en Biotecnología, Universidad Autónoma del Estado de Morelos, Cuernavaca, Morelos, Mexico; 2 Facultad de Farmacia, Universidad Autónoma del Estado de Morelos, Cuernavaca, Morelos, Mexico; 3 Laboratorio de Química Orgánica, Unidad de Biotecnología, Centro de Investigación Científica de Yucatán, Mérida, Yucatán, Mexico

**Keywords:** Hairy roots, *Lepechinia caulescens*, Ursolic acid, Oleanolic acid, Rosmarinic acid, Elicitation, Methyl jasmonate, Sucrose, *Agrobacterium rhizogenes*

## Abstract

**Background:**

Ursolic (UA), oleanolic (OA) and rosmarinic (RA) acids are bioactive metabolites found in *Lepechinia caulescens* that have generated interest for their health benefits, which include antimicrobial, antioxidant, antimutagenic, gastroprotective, antidiabetic, antihypertensive and anti-inflammatory properties, among others. To date, very few attempts have been made to evaluate the potential for simultaneous production of these bioactive compounds, using a biotechnological approach. Hairy root cultures offer a biotechnology approach that can be used to study the factors affecting the biosynthesis and the production of UA, OA and RA. In the current study, we established hairy root cultures of *L. caulescens* and evaluated the effect of sucrose on biomass accumulation, and the effect of different concentrations and times of exposure of methyl jasmonate (MeJA), on the accumulation of UA, OA and RA.

**Methods:**

Leaves from plants of *L. caulescens* were inoculated with *Agrobacterium rhizogenes* strain ATCC 15834*.* PCR of* rolB* gene confirmed the transgenic nature of hairy roots. Hairy roots were subcultured in semisolid MSB5 medium, supplemented with 15, 30, 45 or 60 g/L sucrose and after 4 weeks, dry weight was determined. The accumulation of UA, OA and RA of wild plants and hairy roots were determined by HPLC. Finally, the hairy roots were treated with 0, 100, 200 and 300 *µ*M of MeJA and the content of bioactive compounds was analyzed, after 24, 48 and 72 h.

**Results:**

High frequency transformation (75%) was achieved, using leaf explants from axenic seedlings, infected with *A. rhizogenes*. The hairy roots showed an enhanced linear biomass accumulation, in response to the increase in sucrose concentration. The hairy root cultures in MSB5 medium, supplemented with 45 g/L sucrose, were capable to synthesizing UA (0.29 ± 0.00 mg/g DW), OA (0.57 ± 0.00 mg/g DW) and RA (41.66 ± 0.31 mg/g DW), about two, seven and three times more, respectively, than in roots from wild plants. Elicitation time and concentration of MeJA resulted in significant enhancement in the production of UA, OA and RA, with treatments elicited for 24 h, with a concentration of 300 *µ*M of MeJA, exhibiting greatest accumulation.

**Conclusion:**

This is the first report on development of hairy root cultures of *L. caulescens*. Future studies should aim towards further improving triterpenes and polyphenolic compound production in hairy roots of *L. caulescens,* for use in the pharmaceutical and biotechnological industry.

## Introduction

The triterpenoids ursolic acid (UA) and oleanolic acid (OA), together with rosmarinic acid (RA), an ester of caffeic acid, are secondary metabolites that have gained considerable importance over the last few decades in the pharmaceutical, cosmetic, food and agrochemical industries, due to their wide range of biological activities which include antimicrobial, antioxidant, antimutagenic, gastroprotective, antidiabetic, antihypertensive and anti-inflammatory properties (Qiang et al., 2011; Estrada-Soto et al., 2012; Rios et al., 2012; Soica et al., 2014; Baranauskaite et al., 2016; Lu et al., 2018; Elufioye & Habtemariam, 2019; Giles-Rivas et al., 2020). These compounds commonly occur in stems, leaves and flowers of the Lamiaceae plant family (Woźniak, Ska̧pska S, Marszałek, 2015). However, production levels in nature can be quite low, are often sporadic, and are highly dependent upon factors such as climate, soil, pests and developmental stage of the source plant (Lu, Tang & Li, 2016). Likewise, their chemical synthesis is complex and costly (Phillips et al., 2006; Salvador et al., 2014). Thus, biotechnology options for the production of UA, OA and RA are very necessary. In a recent study, *Lepechinia caulescens* (Ortega) Epling (Lamiaceae), an herbaceous plant used in Mexican folk medicine has been proposed as an alternative source for the production of UA and OA. This plant has elevated triterpene contents (UA: 16.58 mg/g DW and OA: 1.94 mg/g DW), and its callus cultures also accumulate these bioactive compounds (Vergara et al., 2017). However, the culture of undifferentiated cells or calli often provokes problems such as somaclonal variation that can produce low yields of the metabolites of interest and result in genetic and biochemical instability (Srivastava, Gambhir & Sharma, 2013; Ochoa-Villarreal et al., 2016). In contrast, in some cases, hairy root cultures obtained by transformation mediated by *Agrobacterium rhizogenes*, have been shown to be a better option than undifferentiated cell cultures, for the production of bioactive metabolites (Habibi et al., 2016; Häkkinen et al., 2016). Hairy root culture is one of the most efficient tools offered by plant biotechnology for producing secondary metabolites of important industrial interest, particularly pharmaceutical and cosmetic. One of the main advantages of using hairy root cultures is their stable and fast growth rate, without using growth regulators, as well as their genetic and biochemical stability (Roychowdhury, Halder & Jha, 2017). There are strategies that can be used for the hairy root culture system, e.g., the use of elicitors or stressing agents, which can increase the production of secondary metabolites (Alsoufi et al., 2019; Açıkgöz, 2020). The well known elicitor methyl jasmonate (MeJA) is a phytohormone that contributes to the hyperproduction of secondary plant metabolites, as it plays an essential role in the regulation of specific genes, involved in plant development and defense mechanisms against different biotic and abiotic stresses (Ji et al., 2019; Baek et al., 2020). Although previous studies have examined the effects of MeJA on the production of UA, OA and RA in Lamiaceae family (Ruffoni et al., 2016; Li et al., 2020; Yousefian et al., 2020), few studies, with the exception of *Ocimum basilicum* suspension cultures (Pandey, Singh & Banerjee, 2019), have been designed to explore the feasibility of up-scaling the production of these three bioactive metabolites, using an in vitro culture system. Here, we describe the first protocol for the establishment of hairy root culture of *L. caulescens* and present a comparative analysis of the effects of different concentrations and times of exposure to MeJA on the production of UA, OA, and RA. Our results provide valuable guidance for further basic and applied research, aimed at increasing bioactive metabolites in members of the Lamiaceae family.

## Materials & Methods

### Induction and culture of hairy roots

Wild plants of *L. caulescens* were collected from Morelos State, Mexico. The plant was identified in the HUMO Herbarium, Autonomous University of Morelos, where a voucher specimen (32444) was deposited. Mature seeds were germinated and grown *in vitro* on MS medium, solidified with 3.0 g/L phytagel (Vergara et al., 2017). Hairy roots were induced on sterile 4-week-old leaves. Leaf explants consisted of a petiole and small portions of leaf blade. Prior to infection, the *Agrobacterium rhizogenes* strain ATCC 15834 (American Type Cultures Collection, Manassas, USA), harboring agropine-type plasmid pRi15834 was cultivated on liquid YM medium (Hooykaas et al., 1977) in an orbital shaker at 150 rpm in the dark at 28 °C, until the optical density at *λ* = 600 nm (OD600) reached 0.5.

Twenty explants of each type were inoculated with *A. rhizogenes* by immersing them in bacterial suspension for 1 min, and then blotted on filter paper. Control explants were treated identically, using YM medium without bacteria. Thereafter, the infected explants and controls were placed for 24 h on MSB5 medium containing MS salts (Murashige & Skoog, 1962), B5 vitamins (Gamborg, Miller & Ojima, 1968), 0.1 g/L myo-inositol, 30 g/L sucrose and then solidified with 3.0 g/L phytagel. The medium was adjusted to pH 5.7, before being autoclaved at 121 °C for 25 min.

Following co-cultivation, the inoculated and control explants were washed with sterile water, blotted dry on sterile filter paper and placed on MSB5 medium, supplemented with 400 mg/L cefotaxime and 400 mg/L ceftriaxone, in order to eliminate bacteria. After four weeks in the dark at 25 °C, the roots formed by infected explants were cut off and transferred into Petri dishes containing 30 mL MSB5 media with no growth regulators and maintained in the dark. Each excised fast growing root was propagated as a separate clone or line. Transformation frequency was calculated as a percentage of the number of inoculated explants that formed roots, out of the total number of explants used for transformation. Root tips (∼2 g fresh weight) were harvested from 4-week old hairy root cultures and subcultures were commenced at 4-week intervals, in the same media and in dark conditions.

### DNA extraction and PCR analysis

Total DNA of *L. caulescens* hairy roots and seedlings were extracted using the ZR Plant/Seed DNA MicroPrepTM kit (Zymo Research), following manufacturer’s instructions. The quality and quantity of DNA were analyzed using a Nanodrop (NanoDrop 2000, Thermo scientific). Specific primers for *rolB* were used to detect PCR of transgenes in DNA extracts from roots, present in the T-DNA region of pRi15834 from *A. rhizogenes*. The primer pair for amplifying the *rolB* region was 5′ATGGATCCCAAATTGCTATTCCCCCACGA3′ and 3′TTAGGCTTCTTTCATTCGGTTTACTGCAGC5′ (Ashraf et al., 2015). These yields amplified products of 776 bp. Further hairy root lines were tested for the absence of *A. rhizogenes* contamination, using the primer set; forward primer 5′ATGCCGATCGAGCTCAAGT3′ and reverse primer 3′CCTGACCCAAACATCTCGGCTGCCA5′, which amplified a specific sequence (338 bp) of *virD2* gene, located outside the T-DNA (Haas et al., 1995). The genomic DNA from normal roots obtained from *in vitro* seedlings and DNA of bacteria were used as controls. The PCR mix for each amplification contained Taq Buffer 10X (Vivantis, USA) 2.5 µL, MgCl_2_ (25 mM) 1.6 µL, dTNPs (2 mM) 2 µL, primers 3 µL, Taq polymerase 0.5 µl (Vivantis, USA) 9.4 µL ultrapure water and 1 µL DNA (50 ng/ µL) template, at a final volume of 25 µL. Conditions for cycles consisted of an initial denaturation at 94 °C for 5 min; followed by 42 cycles of 1 min denaturation at 94 °C, annealing at 52.5 °C for 1.5 min, and extension at 72 °C for 2 min. A final extension cycle of 10 min at 72 °C, completed the PCR. PCR products were resolved by electrophoresis, using 0.8% (w/v) agarose gels in TAE buffer (0.5 M EDTA, 80 mM Tris acetate at pH 8.0), stained with ethidium bromide (0.5 µg/mL) and observed under UV light.

### Effect of sucrose concentration on biomass accumulation

One-month old hairy roots (∼2 g fresh weight) of L1 and L5 were inoculated into 250 mL jars, containing 20 mL modified MSB5 medium, supplemented with 15, 30, 45 or 60 g/L sucrose. All other components of MSB5 medium remained unchanged. The hairy roots were incubated in darkness at 25 °C for 4 weeks. Subsequently, roots were dried to a constant weight, in an oven set at 30 °C, to determine dry weight (DW). Each treatment consisted of six replicates. Biomass data were analyzed by linear regressions using STATISTICA 7 software; possibility of significance (*P* < 0.05). The coefficient of determination (*R*^2^) was calculated to establish the strength of linear association between the values.

### Extraction and quantification of ursolic, oleanolic and rosmarinic acids

Powdered samples of plant material were extracted by successive maceration at room temperature with hexane, dichloromethane and methanol, every 72 h on three occasions respectively, at a ratio of 1:10 (w/v). Extracts were filtered using filter paper and then air dried in a fume hood.

Separation of UA, OA and RA was achieved, using an analytical high performance liquid chromatography system (Waters HPLC System) and the Waters XBridge™ C18 column (4.6 mm Å∼75 mm). The triterpenes from dichloromethane extracts were separated by applying a mobile phase of methanol: acidic water (H_3_PO_4_) 83:17 (v/v), at a flow rate of 0.9 mL/min. Separation of RA present in the methanolic extracts was performed by elution, using a linear gradient of solvents A (acetonitrile) and B (0.01% phosphoric acid solution). The gradient was as follows: 5 min 90:10% A:B, 20 min 74:26% A:B and 10 min 90:10% A:B. Elution was performed at a solvent flow rate of one mL/min. Waters 996 photodiode array detector system was used for both triterpene (250 nm) and RA (230 nm) detection. Calibration curves were plotted at six concentrations for each of the standard solutions in methanol, in order to undertake a linear regression analysis of the peak area for each concentration. The corresponding retention time (Rt) and ultraviolet spectrum of the standard and the sample, confirmed the presence of UA (Rt 10.67 min), OA (Rt 10.36 min), and RA (Rt 14.10 min) (Shekarchi et al., 2012; Vergara et al., 2017). Hairy root extracts were spiked with the corresponding standard to confirm identification of UA, OA and RA. Standard references were obtained from Sigma-Aldrich (purity: ≥ 97). All solvents were HPLC grade. Mean values were obtained from three replicates of a pooled sample. Results are presented in milligrams (mg) per gram (g) of DW. All data were analyzed by one-way ANOVA, using STATISTICA 7 software. Significance between means was tested by Tukey’s test (*P* < 0.05).

### Elicitation experiments

Two grams of hairy roots were inoculated into 20 mL MSB5 medium supplemented with 45 g/L in 250 mL jars and maintained in the dark at 25 °C. After 15 days of incubation (exponential phase), MeJA was added individually to each semisolid media to reach a final concentration of 0, 100, 200 and 300 µM. Effects of elicitation on the production of UA, OA and RA in hairy roots were measured after 24, 48 and 72 h grown in these media. The stock solutions of MeJA (95%, Sigma-Aldrich) were prepared by dissolving in 1:99 ethanol:water (v:v) and filtering through a 0.22 µm membrane filter. Ethanol and distilled water were added to control media. Each treatment consisted of five repetitions. All data were analyzed by two-way ANOVA, using STATISTICA 7 software. Significance between means was tested by Tukey’s test (*P* < 0.05).

## Results

### Induction and selection of hairy roots

**Figure 1:**
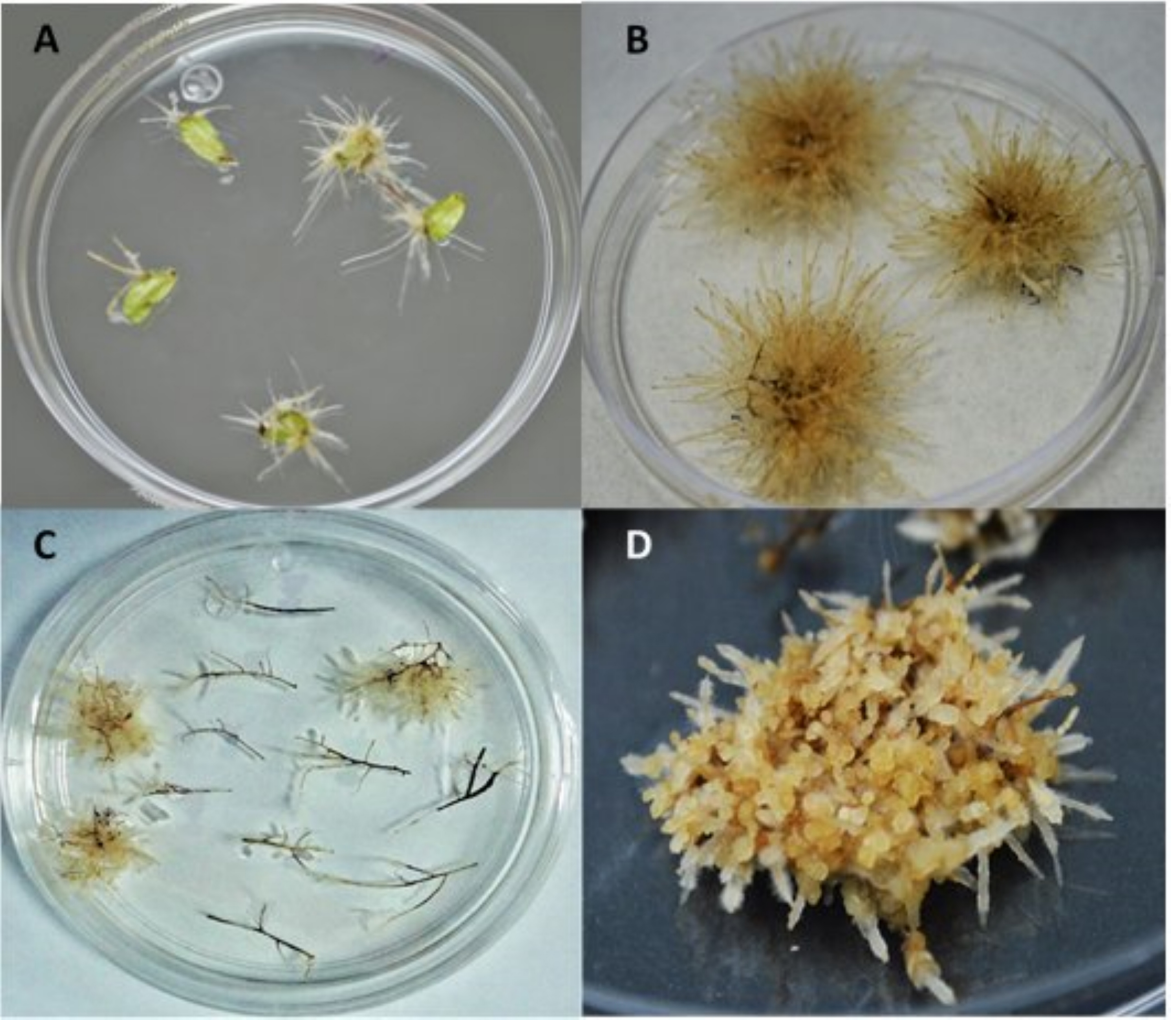
*Agrobacterium rhizogenes* 15834 strain mediated transformation of *L. caulescens*. (A) Hairy roots produced from axenic leaf explants infected with *A. rhizogenes*; (B) individualized and selected transformed root lines; (C) Selection of actively growing hairy roots, non-transformed roots died without any evidence of growth; (D) agglomeration of hairy roots with short lateral branches and spontaneous callus formation.

**Figure 2:**
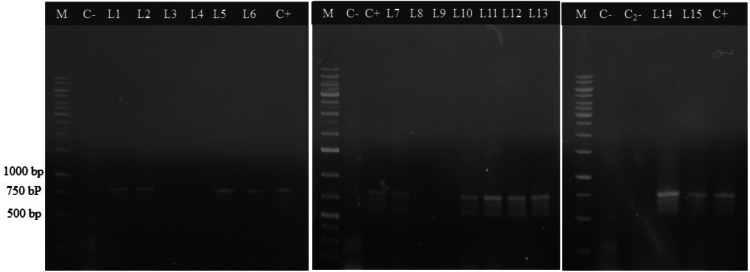
PCR amplification of *rol B* gene fragment. M, molecular size DNA ladder (1 Kb); C+, total DNA from *A. rhizogenes* ATCC15834 strain used as a positive control. C- and C${}_{2}^{-}$, non-infected seedling roots and non-DNA template PCR reaction as negative controls; L1-L15, hairy roots lines.

In this study, hairy roots of *L. caulescens* were generated from axenic leaf explants, infected with *A. rhizogenes* ATCC 15834 (Fig. 1A). Four weeks after infection, hairy roots were induced in almost 75% of the infected leaf blade explants. In contrast, hairy roots did not develop on infected petioles. A total of 56 roots were isolated from infected leaf explants and cultured in MSB5 medium with antibiotic. Whereas the transformed roots showed extensive branching and lack of geotropism, the non-transformed roots turned brown and died, without any evidence of growth (Figs. 1B–1C). After two subcultures in MSB5 medium, the obtained hairy root lines kept their morphology and formed numerous pale yellow branches that began intertwining to form a knot like structure, with short lateral branches and spontaneous callus formation (Fig. 1D). The transgenic nature of eleven hairy root lines was confirmed by PCR analysis when PCR products of DNA isolated from hairy roots, showed the expected 776 bp amplicon corresponding to the *rolB* gene, which was also observed in the positive control (bacterial DNA); alternatively, the PCR product from uninfected *L. caulescens* seedlings could not be amplified (Fig. 2). Ten of the hairy root lines analyzed did not amplify the *virD2* gene fragment indicating the absence of *A. rhizogenes* contamination in these cultures (supplementary material). After 4 subcultures, only L1 and L5 showed satisfactory growth without callus formation and were selected for further study. The other nine hairy root lines formed multiples callus and ceased growth.

### Effect of sucrose concentration on hairy root growth

**Figure 3:**
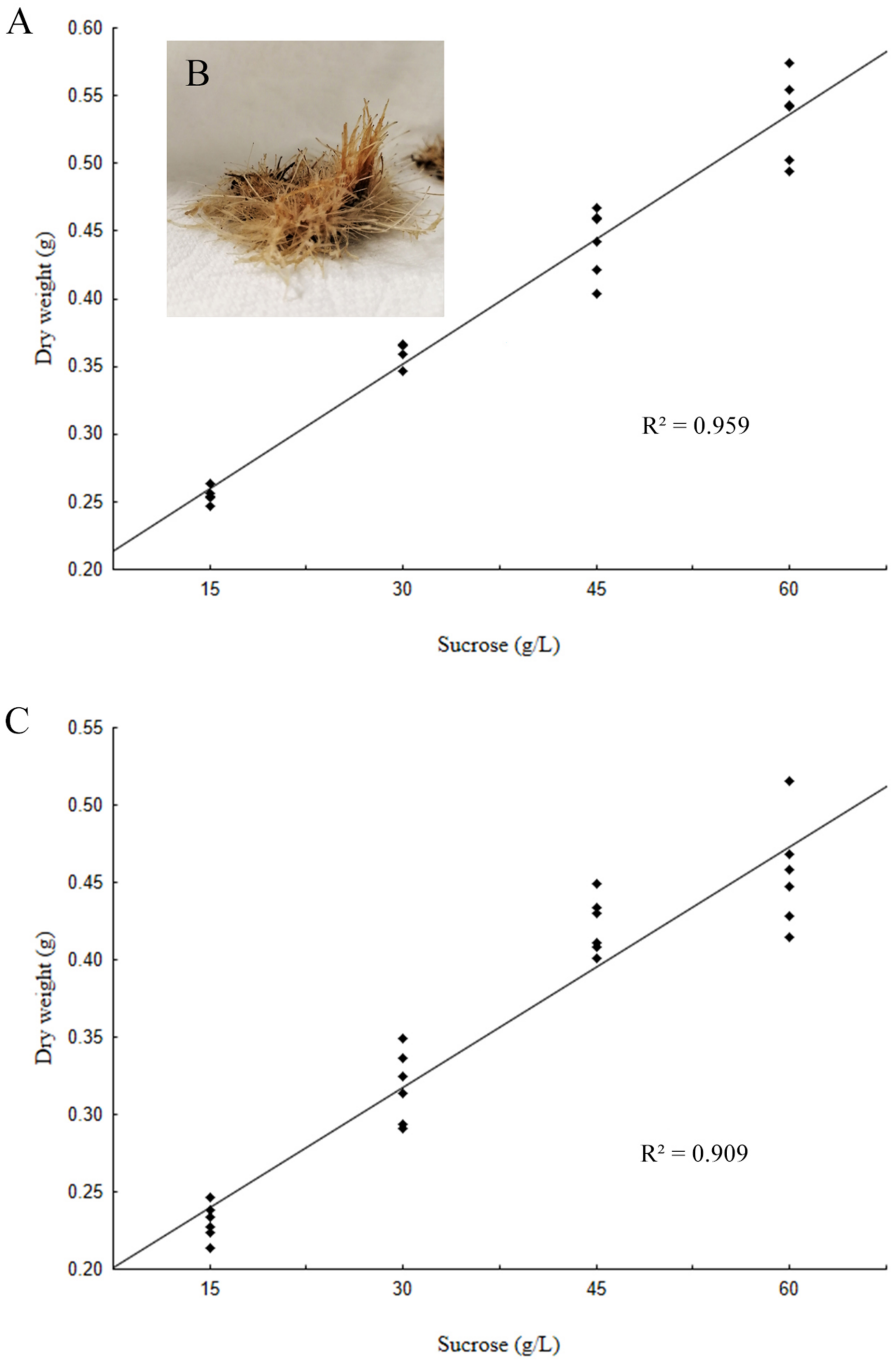
Effect of the variation of sucrose concentration in the MSB5 medium, on the accumulation of biomass of hairy root cultures. (A) L1: *R*^2^ = 0.959, *P* < 0.05; (B) hairy roots of *Lepechinia caulescens* grown in MSB5 medium added with 45 g of sucrose; (C) L5: *R*^2^ = 0.909, *P* < 0.05.

Two lines characterized by hairy root morphology, transgenic nature (detection of *rol* genes) and constant growth rate during 160 days were selected to evaluate the effect of the variation of sucrose concentration in the MSB5 medium on the accumulation of biomass and morphology. As shown in Fig. 3, after 4 weeks in culture, hairy roots showed a proportionate increase in DW, in response to an increase in the concentration of sucrose. Although the highest sucrose concentration treatment (60 g/L) induced browning and thinning of roots, hairy roots grown in MSB5 medium, supplemented with 15, 30 and 45 g/L sucrose, showed less stress symptoms. Noticeably, the hairy roots cultured in media supplemented with 45 g/L of sucrose showed a consistent reduction in formation of calli, were more elongated, thick, and pale yellow (Fig. 3B). Also, both lines have a similar pattern of biomass accumulation (Fig. 3). On the basis of these findings, a culture medium with 45-g/L of sucrose was selected to carry out comparative analysis on the production of bioactive metabolites in hairy root cultures of *L. caulescens* elicited by MeJA.

### HPLC analyses of wild plants and hairy root cultures

The contents of UA, OA and RA in extracts from wild plants were compared with those from two lines of four week old hairy root cultures (Table 1). While, the wild leaves showed a much higher content of three metabolites than the hairy roots, the accumulation of these metabolites in the hairy root cultures was greater than that detected in wild root extracts. Interestingly, RA levels were between 2.9 - to 1.5 times higher in hairy root cultures than in wild roots (14.15 ± 09 mg/g DW), but similar to wild leaves (28.08 ± 0.53 mg/g DW).

### Effect of methyl jasmonate on the content of UA, OA and RA in hairy roots of *L. caulescens*

**Figure 4:**
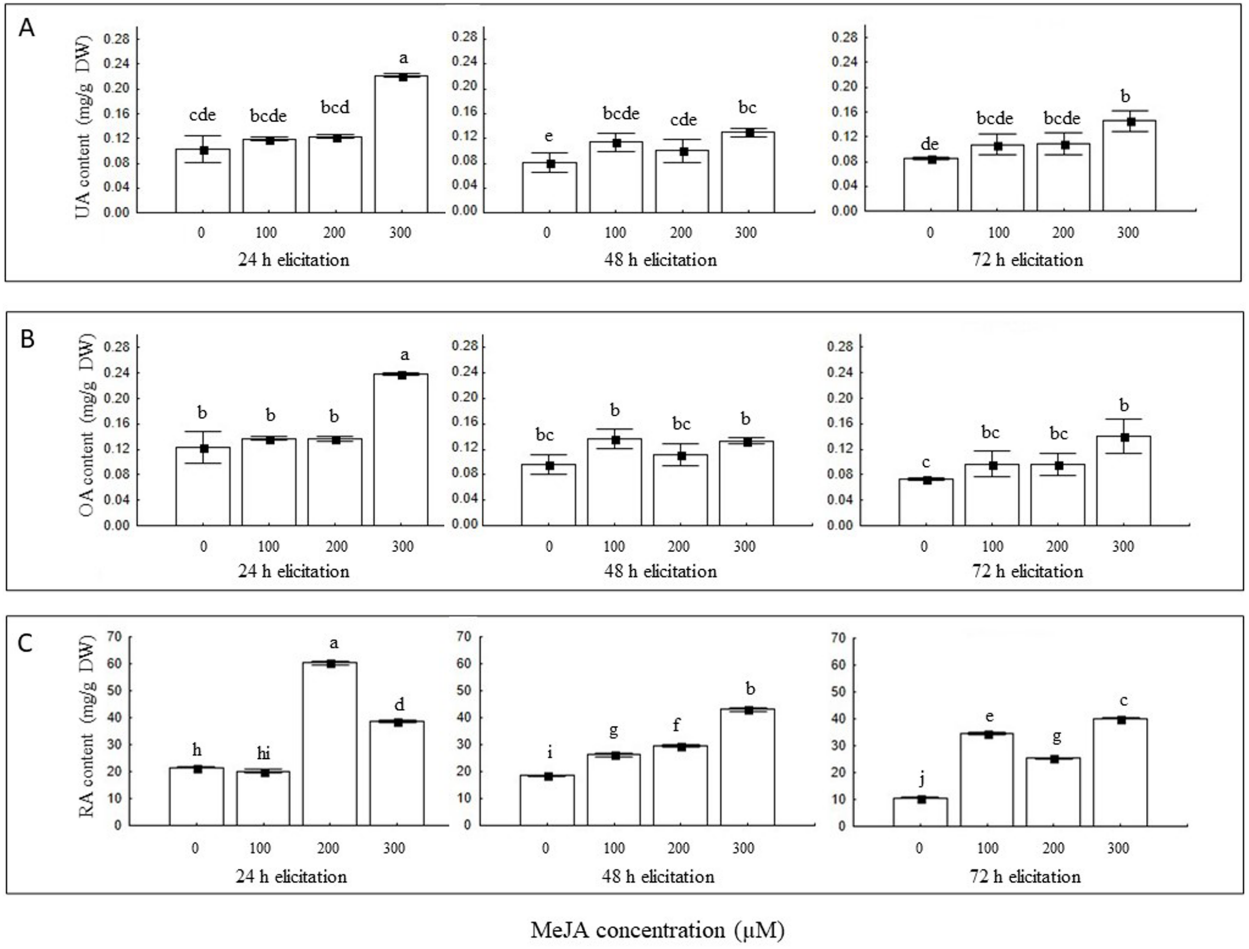
Effect of MeJA concentration and time exposure on ursolic acid (A), oleanolic acid (B), and rosmarinic acid (C) content in hairy roots cultures line 5. Results are the mean of five replicates ± standard deviation. Different letters indicate significant differences by Tukey’s test (*P* < 0.05).

The L5 hairy root line was selected for these evaluations because of its growth stability, absence of calli and superior accumulation of both triterpenes, when compared to L1 line. The production of UA showed significant differences (*F*_6;24_ = 6.581 and *P* < 0.001) between the interaction of MeJA concentration and elicitation time. The highest concentration of UA was obtained by eliciting the cultures for 24 h with 300 *µ* M of MeJA with a production of 0.22 ± 0.00 mg/g DW. Alternatively, the lowest concentration of UA (0.08 ± 0.01 mg/g DW) was obtained in the control treatment (48 h elicitation without MeJA) (Fig. 4). Similar results were obtained for the accumulation of OA; the interactions MeJA concentration and elicitation time showed significant differences (*F*_6;24_ = 6.631 and *P* < 0.001), the highest yield of OA (0.24 ± 0.00 mg/g DW) was obtained by eliciting the hairy roots cultures with a concentration of 300 *µ* M of MeJA, for 24 h; while the lowest concentration of OA (0.07 ± 0.00 mg/g DW) was obtained in the control treatment (72 h elicitation, without MeJA) (Fig. 4). The accumulation of RA was also favored by eliciting cultures with MeJA. Interactions between MeJA concentration and elicitation time showed significant differences (*F*_6;24_ = 1491.0 and *P* < 0.001). The highest accumulation of RA (60.27 ± 0.88 mg/g DW) was obtained by eliciting the cultures with 200 *µ*M of MeJA for 24 h; and the lowest concentration (10.71 ± 0.34 mg/g DW) was obtained in the control treatment (72 h, without MeJA) (Fig. 4).

**Table 1:** Comparative accumulation of ursolic, oleanolic and rosmarinic acids in extracts from hairy roots and wild plants of *L. caulescens*. The results are mean values ± SD of three replicates for each plant material. Different letters mean significant statistical differences between samples according to a one-way ANOVA, followed by the post hoc Tuckeys test for multiple comparison at *p* < 0.05. UA, ursolic acid; OA, oleanolic acid; RA, rosmarinic acid.

Material	UA (mg/g DW)	OA (mg/g DW)	RA (mg/g DW)
Hairy root lines			
L1	0.10 ± 0.00^D^	0.17 ± 0.00^C^	41.66 ± 0.31^A^
L5	0.29 ± 0.00^B^	0.57 ± 0.00^B^	21.60 ± 0.38^C^
Wild roots	0.17 ± 0.00^C^	0.08 ± 0.00^D^	14.15 ± 0.09^D^
Wild leaves	16.58 ± 0.05^A^	1.94 ± 0.05^A^	28.08 ± 0.53^B^

According to statistical analyses, the yield means of the elicitation time factor (*F*_3,24_ = 23.127, *F*_3,24_ = 43.374 and *F*_3,24_ = 695.78 for UA, OA and RA respectively, *P* < 0.001 for all cases) and the concentration of MeJA (*F*_3,24_ = 49.213, *F*_3,24_ = 36.881 and *F*_3,24_ = 4114.9 to UA, OA and RA respectively, *P* < 0.001 for all cases) have a positive effect on the production of UA, OA and RA; with treatments elicited for 24 h with a concentration of 300 µM of MeJA, showing greatest accumulation.

## Discussion

*L. caulescens* represents a suitable plant species for the establishment of plant tissue cultures to produce UA, OA and RA, as valuable additive and bioactive compounds for pharmaceutical, cosmetic and food industries (Perez-Hernandez et al., 2008; Vergara et al., 2017). Hairy roots induced by *A. rhizogenes* provide an attractive alternative system to classic technologies to produce bioactive compounds, because of their higher degree of genetic and biochemical stability (Tabar, Moieni & Monfared, 2019). In this investigation, hairy root cultures of *L. caulescens* were established from leaf explants, using *A. rhizogenes* ATCC 15834. This strain is widely used for the production of important bioactive compounds from many aromatic and medicinal plants of the Lamiaceae family, such as *Prunella vulgaris* (Ru et al., 2016), *Dracocephalum kotschyi* (Nourozi et al., 2019), *Nepeta teydea* (Fraga et al., 2017) *Pogostemon Cablin* (He-Ping et al., 2011) and *Origanum vulgare* (Habibi et al., 2016). In most of these plant species and in *L. caulescens,* leaf explants stand out as the best tissue for transformation. Results from this investigation indicate that petiole explants were not susceptible to transformation by *A. rhizogenes*. These differences between leaf and petiole explants may be due to different tissue sensitivity to *Agrobacterium* strain, variations in T-DNA integration efficiency and varying survival rates of transgenic cells after treatment. The efficiency of *A. rhizogenes* strains to induce for hairy roots can vary greatly. It is well documented that success in *Agrobacterium*-mediated plant genetic transformation processes relies on various factors, such as bacterial strains, plasmid, age of explants, and capacity of target plant tissues for infection (Karami et al., 2009).

The transgenic nature of hairy root lines of *L. caulescens* was confirmed by PCR amplification of *rolB* gene of T-DNA. The *rolB* gene is crucial for the *de novo* induction of meristems and elongation of hairy roots (Mauro, Costantino & Bettini, 2017; Fathi, Mohebodini & Chamani, 2019). In the present study, all the selected hairy root lines exhibited a densely clumped growth, short branches and rapid plagiotropic growth on plant growth regulator free medium. This may be due to the lack of apical dominance here, owing to the expression of genes on the T-DNA.

The *L. caulescens* hairy roots kept in darkness are non autotrophic and thus rely solely on an external carbon source for their growth and metabolic requirements. Sucrose is the good carbon source for the cultivation of *in vitro* plant tissues, due to its effective absorption through the cell membrane (Ptak et al., 2020), which can provide energy, building blocks and osmotic pressure (Wiktorowska, Długosz & Janiszowska, 2010), but also affect hormone biosynthesis and secondary metabolism (Thompson et al., 2017). It can particularly affect the growth and development of hairy roots, just as it was observed in this study. However, and even though increased sucrose concentration in culture medium caused enhanced accumulation of hairy root biomass and decreased callus formation a steep increase in the browning of roots after 21 days of culture in 60 g/L of sucrose was observed; this can be associated with osmotic stress and loss of culture viability. Thus, the results from this study revealed that supplementing the medium with 45 g/L sucrose, improves biomass accumulation and morphology of *L. caulescens* hairy roots*.* The concentrations of between 3%–5% sucrose have frequently been reported as optimal for biomass accumulation and production of secondary metabolites from adventitious roots and hairy root cultures, whereas higher concentrations of sucrose have an inhibitory effect on plant tissue culture growth (Vazquez-Flota et al., 1994; Baque et al., 2012; Praveen & Murthy, 2012). Similarly, the supplementation of cultures with sucrose has also been reported to influence the growth and development of hairy root cultures of *Psoralea corylifolia* (Shinde, Malpathak & Fulzele, 2010), *Hypericum perforatum* (Cui et al., 2010), *Azadirachta indica* (Srivastava & Srivastava, 2012) and *Daucus carota* (Mukherjee et al., 2019).

The results presented here indicate that hairy root cultures of *L. caulencens* have the ability to synthesize and accumulate UA, OA and RA. However, there were apparent differences in their hairy root line content, when cultured under the same conditions. These differences between the L1 and L5 transgenic lines may be caused by varying expression levels of *rol* genes that possibly refer to plant genome variations at the T-DNA insertion site and in the copy number. Several authors have reported that the location and copy number of *rolB* and *rolC* genes, alone or in combination with each other, can affect hairy root growth and secondary metabolite production (Shkryl et al., 2008; Ahlawat et al., 2014; Dilshad et al., 2015; Matvieieva et al., 2020). Supplementation with MeJA significantly increased the amount of UA, OA and RA in the hairy roots of *L. caulencens*. This elicitor modulates key enzymes involved in the biosynthesis of triterpenes and phenolic compounds (Misra et al., 2014). Wang et al. (2017) found that the addition of MeJA to cell cultures of *Achyranthes bidentata* in suspension*,* up-regulated the mRNA expression level of 3-hydroxy-3-methylglutaryl coenzyme A reductase gene (*HMGR*), encoding a key enzyme to provide mevalonate for biosynthesis of triterpene acids. Likewise, Misra et al. (2014) and Misra et al. (2017) found that *β*-amyrin synthase (*AS*) and two cytochrome P450 monooxygenases genes, increased their expression levels in *Ocimum basilicum,* when elicited with 250 µM MeJA, thus contributing to the accumulation of OA and UA. It has been observed that MeJA can also elevate the expression of the key genes involved in the biosynthesis of RA, such as phenylalanine ammonia-lyase gene (*PAL*), cinnamate 4-hydroxylase (*C4H*) and 4-coumarate: coenzyme-A (CoA) ligase (*4CL*), tyrosine aminotransferase (*TAT*), 4-hydroxyphenylpyruvate reductase (*HPPR*) and RA synthase (Kim et al., 2013; Xing et al., 2018; Fatemi et al., 2019).

Several studies have shown that the effects of MeJA treatments may vary depending on plant species or specific secondary metabolite, as mentioned above. It has been demonstrated that elicitor concentration and exposure duration exert a stimulatory effect on certain plant systems, but result in null activity when applied to others, reflecting different sensibilities on the part of the molecular components involved in elicitation (Vasconsuelo & Boland, 2007; Verma et al., 2014). In effect, the results from this study clearly demonstrate that the accumulation of ursolic, oleanolic and rosmarinic acids depends on the concentrations of MeJA applied, and duration of exposure to the elicitor. Among the various MeJA treatments, 300 µM MeJA for 24 h was suitable for increasing the production of UA and OA, whereas 200 µM for 24 h of this elicitor was the optimum condition for improving RA production in *L. caulencens* hairy roots. Interestingly, when exposure time was increased to 48 or 72 h, the MeJA did not promote accumulation of triterpenes. These results concur with those reported by (Sharan, Sarin & Mukhopadhyay, 2019), who found variation in the yield of UA in *Ocimum tenuiflorum* hairy root cultures, depending on the concentration of MeJA (0, 30, 60 and 120 mg/L) and exposure times (4, 8 and 12 days). This effect on the part of MeJA was also observed in terms of OA yield from the cell cultures of *Achyranthes bidentata,* treated with 0.2 mM MeJA for 24, 48 and 72 h (Wang et al., 2017) and for the accumulation of RA in the hairy root cultures of *Coleus forskohlii,* exposed to MeJA (0.1, 0.5 and 1.0 mM) for between 1 and 7 days (Li et al., 2005). As concentration and exposure time to the elicitor are plant specific, the results described in this paper may contribute to future molecular studies, designed to analyze the biosynthesis pathway of these valuable pharmaceutical compounds.

## Conclusions

In summary, this is the first report to document the successful establishment of *L. caulescens* hairy root cultures. The hairy roots grown in MSB5 medium, supplemented with 45g/L sucrose, have the ability to synthesize and accumulate significantly higher UA, OA and RA concentrations than the wild plant. Furthermore, this study clearly demonstrated that the accumulation of these bioactive metabolites was dependent on the MeJA concentrations used, and duration of exposure to the elicitor. Future studies should aim to identify the regulatory factors that control the biosynthetic pathways of triterpenes and phenolic compounds in hairy roots of *L. caulescens*.

##  Supplemental Information

10.7717/peerj.11279/supp-1 Supplemental Information 1 Gel electrophoresis of PCR products of virD2 gene M, DNA marker, C-, DNA template (non-inoculated seedling); Sample L1and L15, genomic DNA of hairy root lines; C+, plasmid DNA

10.7717/peerj.11279/supp-2 Supplemental Information 2 Raw table for figure 3

10.7717/peerj.11279/supp-3 Supplemental Information 3 HPLC chromatogram of standards of ursolic acid (UA) and oleanolic acid (OA)

10.7717/peerj.11279/supp-4 Supplemental Information 4 HPLC chromatogram UA and OA HPLC chromatogram of *Lepechinia caulescens* hairy roots (1) and wild plants (2) samples for detecting ursolic and oleanolic acid.

10.7717/peerj.11279/supp-5 Supplemental Information 5 HPLC chromatogram RA HPLC chromatogram of *Lepechinia caulescens* hairy roots (1), wild plants (2) and standard (3) samples of rosmarinic acid.

10.7717/peerj.11279/supp-6 Supplemental Information 6 Raw table for figure 4

## Additional Information and Declarations

### Data Availability

The following information was supplied regarding data availability: Raw data are available as a Supplemental File.
