# Supplementary figures and images for: Methyl jasmonate enhances ursolic, oleanolic and rosmarinic acid production and sucrose induced biomass accumulation, in hairy roots of Lepechinia caulescens
Source: PeerJ. 2021 Apr 27;9:e11279. doi: 10.7717/peerj.11279 (PMC8086586; doi:10.7717/peerj.11279)

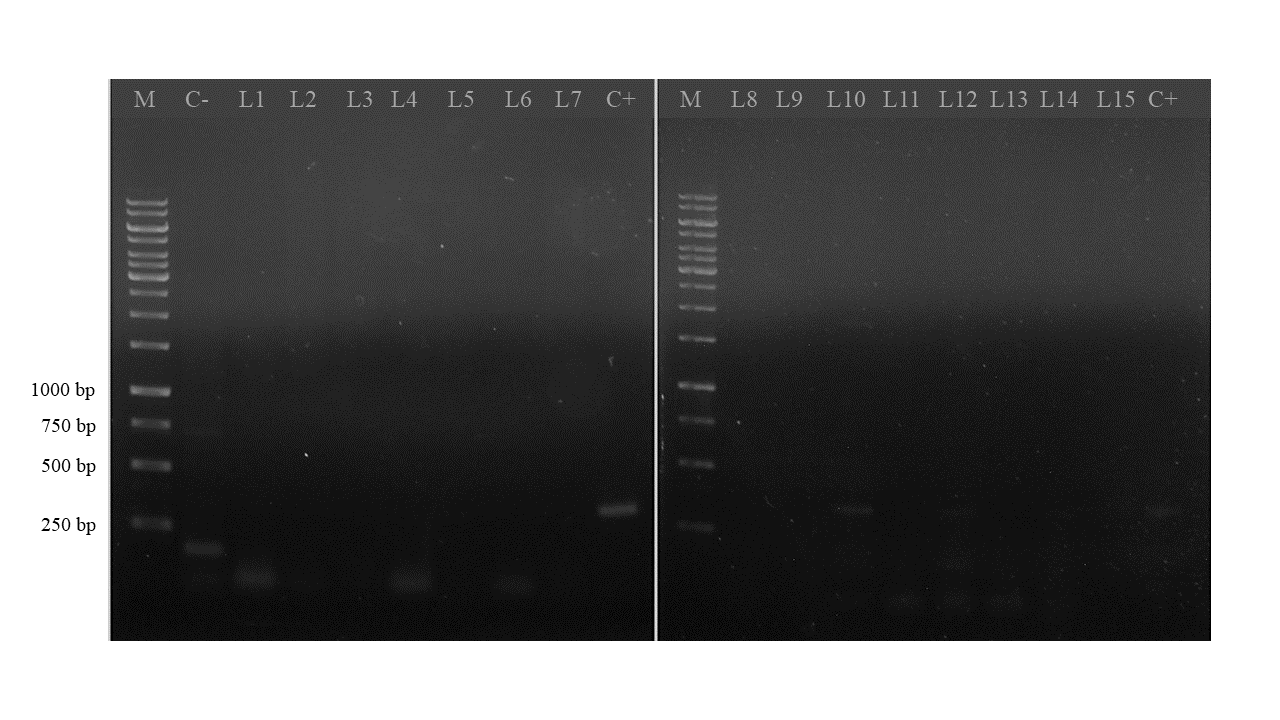

Supplement: Supplemental Information 1 — M, DNA marker, C-, DNA template (non-inoculated seedling); Sample L1and L15, genomic DNA of hairy root lines; C+, plasmid DNA [file peerj-09-11279-s001.png]

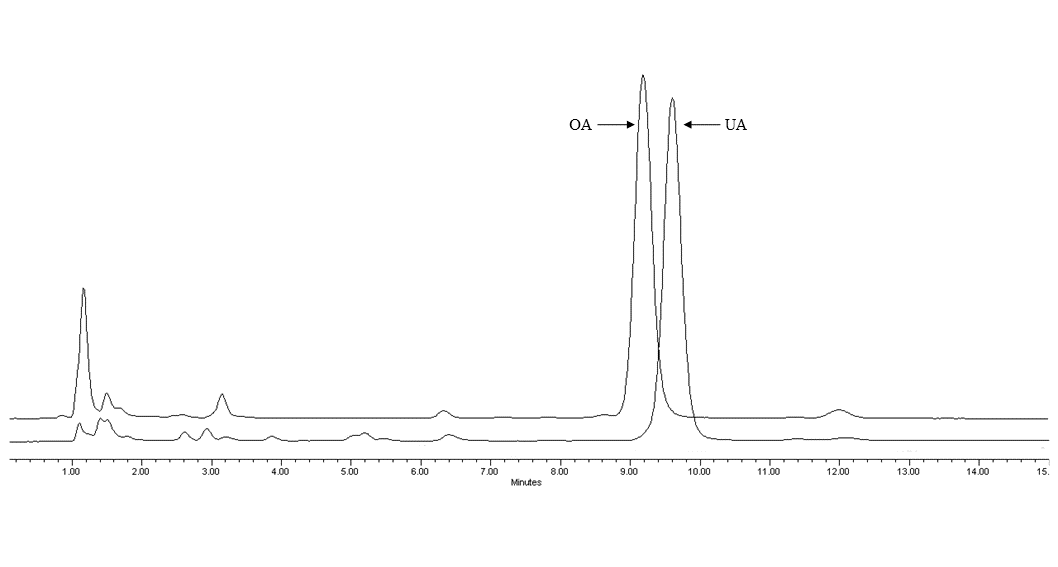

Supplement: Supplemental Information 3 [file peerj-09-11279-s003.png]

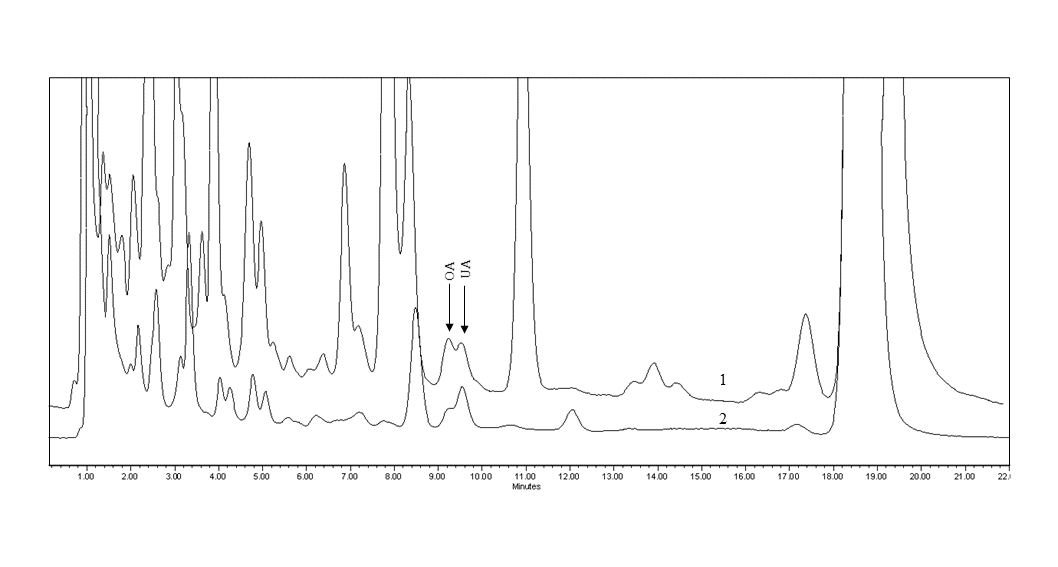

Supplement: Supplemental Information 4 — HPLC chromatogram of Lepechinia caulescens hairy roots (1) and wild plants (2) samples for detecting ursolic and oleanolic acid. [file peerj-09-11279-s004.png]

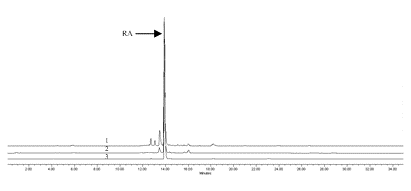

Supplement: Supplemental Information 5 — HPLC chromatogram of Lepechinia caulescens hairy roots (1), wild plants (2) and standard (3) samples of rosmarinic acid. [file peerj-09-11279-s005.png]
